# Prediction of surgical benefit in gastric cancer patients with peritoneal metastasis treated with hyperthermic intraperitoneal chemotherapy

**DOI:** 10.1007/s13304-024-01989-y

**Published:** 2024-10-04

**Authors:** Shiyang Jin, Yuzhe Wei, Qiancheng Wang, Yuming Ju, Zeshen Wang, Qingqing Cheng, Zhenglong Li, Xirui Liu, Kuan Wang

**Affiliations:** Department of Gastrointestinal Surgery, Harbin Medical University Cancer Hospital, No. 150, Haping Road, Nangang District, Harbin, 150081 Heilongjiang China

**Keywords:** Cytoreductive surgery, Gastric cancer, Hyperthermic intraperitoneal chemotherapy, Nomogram, Peritoneal metastasis

## Abstract

The objective is to evaluate whether gastric cancer patients with peritoneal metastasis can benefit from surgery through a comprehensive analysis of different clinical factors and perioperative treatment methods. A total of 135 gastric cancer patients with peritoneal metastasis were treated with Hyperthermic intraperitoneal chemotherapy (HIPEC). Patients were divided into either training group (without surgery, *n* = 90) or test group (with surgery, *n* = 45). A nomogram was constructed based on significant prognostic factors. The patients were divided into high- and low-risk groups using a nomogram. Overall survival were then compared according to whether surgery was performed in both groups. Alpha-fetoprotein (AFP), complications, conversion chemotherapy, and postoperative chemotherapy were significantly associated with overall survival (*p* < 0.05). A nomogram was constructed using the above four factors and validated using the test set. The area under the curve (AUC) of the model was 0.752 (95% CI 0.525–978). In the group that did not undergo surgery, the median survival times for the high-risk and low-risk groups were 7 and 11 months, respectively. In the surgery group, the median survival times for the high-risk and low-risk groups were 11 and 19 months, respectively. The difference was statistically significant (*p* < 0.0001). The four-factor nomogram can accurately predict high-risk and low-risk populations. Our findings suggest that cytoreductive surgery combined with HIPEC can improve the survival time of patients in both groups.

**Supplementary Information:**

The online version contains supplementary material available at 10.1007/s13304-024-01989-y.

## Introduction

Gastric cancer is a malignant tumor with the fifth highest mortality rate worldwide, and China has one of the highest incidences of gastric cancer globally [1, 2]. Peritoneal metastasis is a common form of metastasis in patients with gastric cancer and the proportion of patients with peritoneal metastasis gradually increases as the disease progresses. Complications such as malignant ascites and intestinal obstruction caused by peritoneal metastasis are important causes of death in gastric cancer patients [3]. Early studies have classified the presence of peritoneal metastasis under stage IV gastric cancer. Previous reports have shown that surgical treatment has a limited effect in gastric cancer patients with peritoneal metastasis. However, with the recent advancement of research, many experts suggested that some peritoneal metastasis is more like a locally advanced cancer, and consequently, surgery may be of benefit to this part of patients [4].

In recent years, hyperthermic intraperitoneal chemotherapy (HIPEC) has made rapid progress in the treatment of cancer. Being a local treatment, HIPEC can remain unaffected by the peritoneal barrier, thereby directly killing abdominal tumor cells. HIPEC plays an important role in ovarian and colorectal cancers. In addition, some studies have shown that HIPEC combined with chemotherapy can significantly prolong the survival of gastric cancer patients with peritoneal metastasis compared to chemotherapy alone [5–7]. Previous studies have also reported that patients who have undergone intraperitoneal (without hyperthermic) plus systemic chemotherapy and achieved R0 resection have significantly longer survival times than patients who have not undergone surgery, thus suggesting that some patients with peritoneal metastases may benefit from surgery [8]. Other studies suggested that HIPEC combined with cytoreductive surgery (CRS) can significantly prolong the survival time of gastric cancer patients with peritoneal metastasis compared with cytoreductive surgery alone [9–11]. Therefore, CRS combined with HIPEC has gradually become a great treatment method in gastric cancer with peritoneal metastasis. However, some patients who undergo surgery survive for less than 6 months even after HIPEC [12]. For these patients, surgery may not confer any survival benefit and may instead increase the risk of various complications and costs associated with surgery. Whether or not surgery should be performed in these patients remains controversial [13, 14]. To date, no studies have examined in detail the benefits of surgery in HIPEC patients with different clinical factors.

Given the complex condition of gastric cancer with peritoneal metastasis, traditional multivariate analysis cannot accurately identify all clinical risks. To address this problem, this study aimed to develop a nomogram to fully assess the survival risk of patients. In recent years, nomograms have emerged as an efficient statistical tool. It can integrate the influence of multiple factors and calculate the survival probability of each patient through intuitive graphics. It is based on the regression coefficient to specify the scoring criteria, and for each patient, a total score calculated by comparison with the probability of outcome can be used to obtain survival prediction [15–18]. The current study was designed to stratify peritoneal metastases from gastric cancer patients who underwent HIPEC by establishing a reliable nomogram and comparing the surgical benefits in high- and low-risk populations.

## Patients and methods

### Study population

**Fig. 1:**
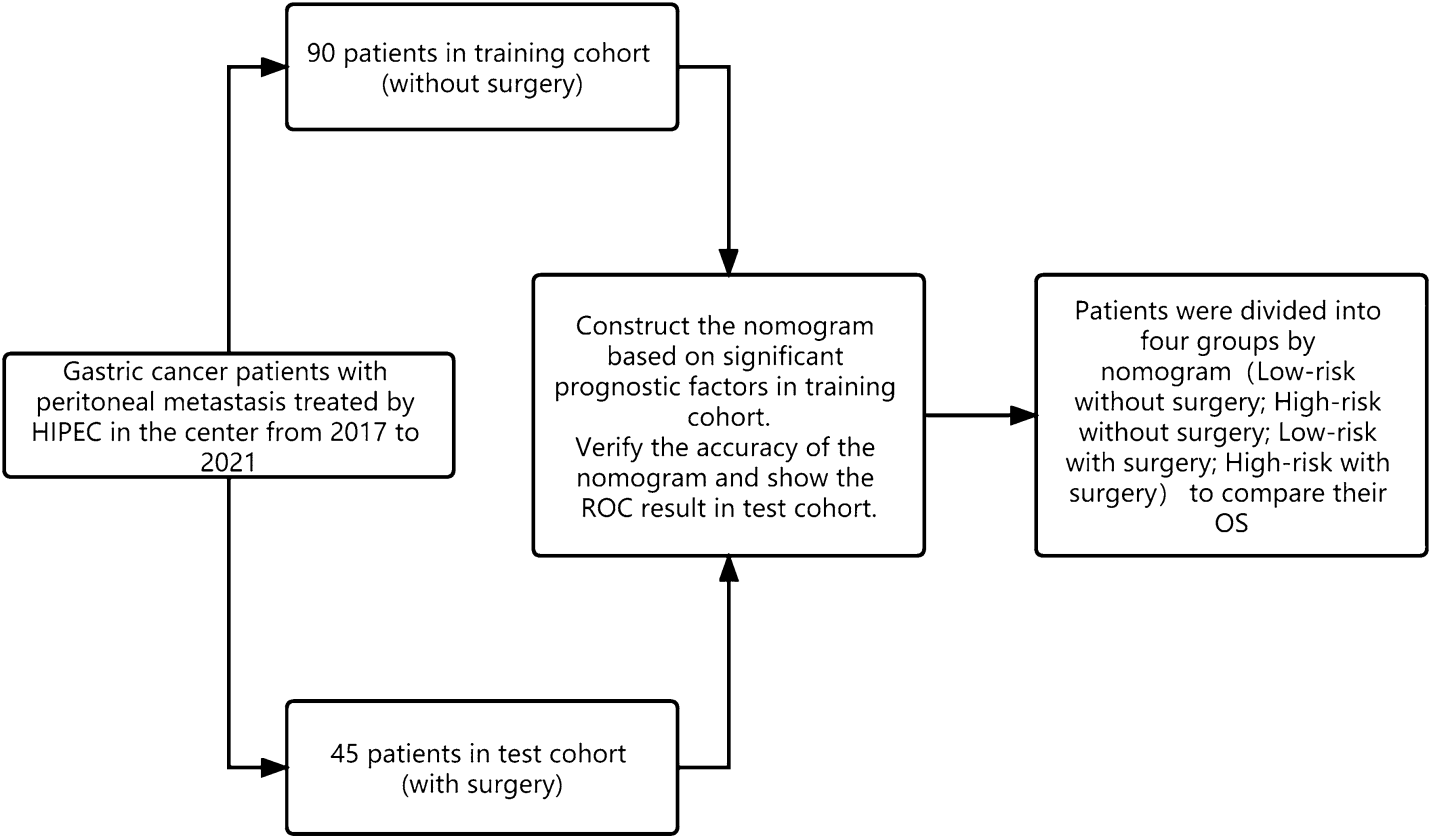
The flowchart of the article

This study evaluated gastric cancer patients with peritoneal metastasis treated with HIPEC at Harbin Medical University Cancer Hospital between 2017 and 2021. The inclusion criteria were as follows: (1) age > 18 years; (2) Eastern Cooperative Oncology Group performance status (ECOG-PS) score of 0–1; (3) pathological diagnosis of gastric cancer or gastroesophageal junction adenocarcinoma; (4) peritoneal metastasis diagnosed by laparoscopic exploration or CT; (5) at least one HIPEC; and (6) no other primary tumor in the past 5 years. The exclusion criteria were as follows: (1) chronic diseases that could affect toleration of surgery and chemotherapy; (2) ECOG-PS score ≥ 2; (3) no HIPEC performed in our hospital; (4) severe liver and kidney dysfunction; (5) other distant metastases at diagnosis; and (6) previous history of another malignancy (Fig. 1).

### HIPEC catheterization

During the HIPEC treatment, four drainage tubes, composed of two perfusion tubes and two outflow tubes, were placed on the axillary front plane. The perfusion tubes were placed on the upper abdomen, whereas the outflow tubes were placed on both sides of the pelvic floor. The perfusion temperature setting was 43 °C, the perfusion time was 60 min, and the perfusion speed was at least 400 mL/min. The amount of perfusion fluid was determined by filling the patient’s abdominal cavity with fluid and establishing a smooth circulation of approximately 3000–5000 mL. HIPEC was carried out at 43 ± 0.1 °C with paclitaxel (75–100 mg/m^2^) as the chemotherapeutic agent. We recommend that HIPEC be conducted on days one, three, and five, respectively. After treatment, approximately 1000 mL of perfusion fluid remained in the abdominal cavity, which was gradually drained through the drainage tube.

### Cytoreductive surgery

We would operate only if at least one of the following criteria was met:

1. The patient and family strongly demand surgery.
2. Significantly fewer peritoneal metastases on secondary laparoscopic exploration.
3. PCI ≤ 6.
4. The doctor is confident that the CC0-CC1 CRS will be performed.

Distal or total gastrectomy was performed according to the size and location of the primary focus, and D2 lymph node dissection was performed concurrently. If the tumor invaded the surrounding organs, such as the small intestine, colon, and pancreas, the affected organs were resected. The metastasis visible to the naked eye was also resected to the greatest extent possible. If ovarian implantation was observed, the ovary was removed.

Patients with peritoneal metastasis after laparoscopic exploration and direct CRS were treated with HIPEC within 48 h postoperatively, and chemotherapy was administered after HIPEC. The SOX regimen (oxaliplatin 100 mg/m^2^ on day one, with S-1 capsules twice daily for 14 consecutive days) was used in most chemotherapy protocols, but other chemotherapy regimens were allowed. For patients with peritoneal metastasis who did not undergo surgery after the initial laparoscopic exploration, catheterization tubes were placed at the time of exploration, followed by HIPEC. Chemotherapy was administered after the HIPEC. If the patients underwent laparoscopic exploration again after chemotherapy or cytoreductive surgery, chemotherapy was defined as conventional chemotherapy. If chemotherapy was continued after surgery, subsequent chemotherapy is defined as postoperative chemotherapy. If the patient no longer underwent any surgery, the chemotherapy was defined as direct postoperative chemotherapy.

### Data collection and follow-up

Survival data were obtained from patient records, outpatient treatment, and telephone follow-ups. Owing to the short survival time, patients were followed up once a month for two years and then every 3 months thereafter. Overall survival (OS) was calculated from the first day of diagnosis to the date of death or final follow-up (November 20, 2021). The patients were followed-up for an average of 9.7 months (range 2–58 months). The baseline characteristics and survival-related factors were summarized and statistically analyzed.

### Statistical analyses

All patients were divided into two groups based on whether or not they underwent surgery: training group (without surgery) and test group (with surgery). Continuous variables were analyzed using the *t*-test or Mann–Whitney *U* test, while categorical variables were analyzed using the *χ*^2^ test or Fisher’s exact test. We used three different statistical methods (univariate analysis, best subsets regression [BSR], and least absolute shrinkage and selection operator [Lasso]) to analyze clinical factors and treatment options associated with overall survival (OS). In the univariate analysis, we selected factors with *p* < 0.05. In the BSR regression analysis, we selected the model factors with the largest component of *R*^2^. In the Lasso analysis, we selected the model factors with the smallest mean square error (MSE). A multivariate Cox regression analysis was performed for the three methods groups. Stepwise backward variable removal was applied to multivariate models to identify the most accurate and parsimonious set of predictors. We used the Akaike information criterion (AIC) and area under the curve (AUC) within the Cox regression model of the training cohort to compare the performance of the different prognostic systems. Smaller AIC values and larger AUC values represent better optimistic prognostic stratification. Each variable in the nomogram has a corresponding weighted score and the sum of the weighted scores was associated with OS. The AUC of the test cohort was used to measure the discriminatory ability of the different prognostic systems.

Patients in the training and test sets were assigned values and were cut off from the receiver operating characteristic (ROC) curve of the training set. Patients were divided into high-risk without surgery, low-risk without surgery, high-risk with surgery, and low-risk with surgery groups based on their cut-off values. Survival rates were calculated using the Kaplan–Meier method and compared using the log-rank test. Statistical significance was set at *p* < 0.05. Finally, KM curves of OS were constructed to assess the benefits of surgery in the different groups and were compared to the PCI group results. All data were processed using R software (version 4.1.3).

## Results

### Patient characteristics

A total of 135 patients were included in this study. The training group consisted of 90 patients with a mean age of 53.8 years, while the test group consisted of 45 participants with a mean age of 54.9 years. Age, sex, cT stage, tumor site, type of surgery, and chemotherapy were not significantly different between the training and internal validation sets. Due to a certain bias in the treatment process, patients undergoing surgery tended to have relatively mild disease; therefore, there was a significant difference between the cN stage and peritoneal metastasis index(PCI) groups (*p* < 0.01). Table 1 shows the data of clinical-related factors of patients in the training and test groups (Table 1, Table S1).

**Table 1:** Clinicopathologic characteristics of the patients

Category	Training cohort (without surgery)	Test cohort (with surgery)	*p*
	*N* = 90	*N* = 45	
Age (mean (SD))	53.856 (11.414)	54.956 (10.445)	0.5883
BMI (mean (SD))	22.310 (3.100)	21.867 (3.761)	0.4678
Sex (%)			0.2937
Male	56 (62.22)	23 (51.11)	
Female	34 (37.78)	22 (48.89)	
Pathology (%)			0.7581
Well-moderately differentiated adenocarcinoma	4 (4.44)	1 (2.22)	
Poorly differentiated adenocarcinoma	68 (75.56)	36 (80.00)	
Signet-ring cell carcinoma	18 (20.00)	8 (17.78)	
Tumor site (%)			0.0633
Upper	15 (16.67)	3 (6.67)	
Middle	29 (32.22)	23 (51.11)	
Lower	46 (51.11)	19 (42.22)	
cT (%)			0.9587
T3	9 (10.00)	5 (11.11)	
T4a	70 (77.78)	34 (75.56)	
T4b	11 (12.22)	6 (13.33)	
cN (%)			< 0.0001
N0-1	12 (13.33)	12 (26.67)	
N2	35 (38.89)	33 (73.33)	
N3	43 (47.78)	0 (0.00)	
cM (%)			0.0498
M0	0 (0.00)	2 (4.44)	
P1	86 (95.56)	43 (95.56)	
M1	4 (4.44)	0 (0.00)	
PCI (%)			< 0.0001
< 6	14 (15.56)	23 (51.11)	
7–14	19 (21.11)	13 (28.89)	
> 14	57 (63.33)	9 (20.00)	
Conversion chemotherapy (%)			0.1189
No	82 (91.11)	36 (80.00)	
Yes	8 (8.89)	9 (20.00)	
Postoperative chemotherapy (%)			0.4107
No	30 (33.33)	19 (42.22)	
Yes	60 (66.67)	26 (57.78)	
Yes	4 (4.44)	5 (11.11)	
OS	10 (8–11)	13 (10–NA)	0.0221

### Establishment and verification of nomogram

**Fig. 2:**
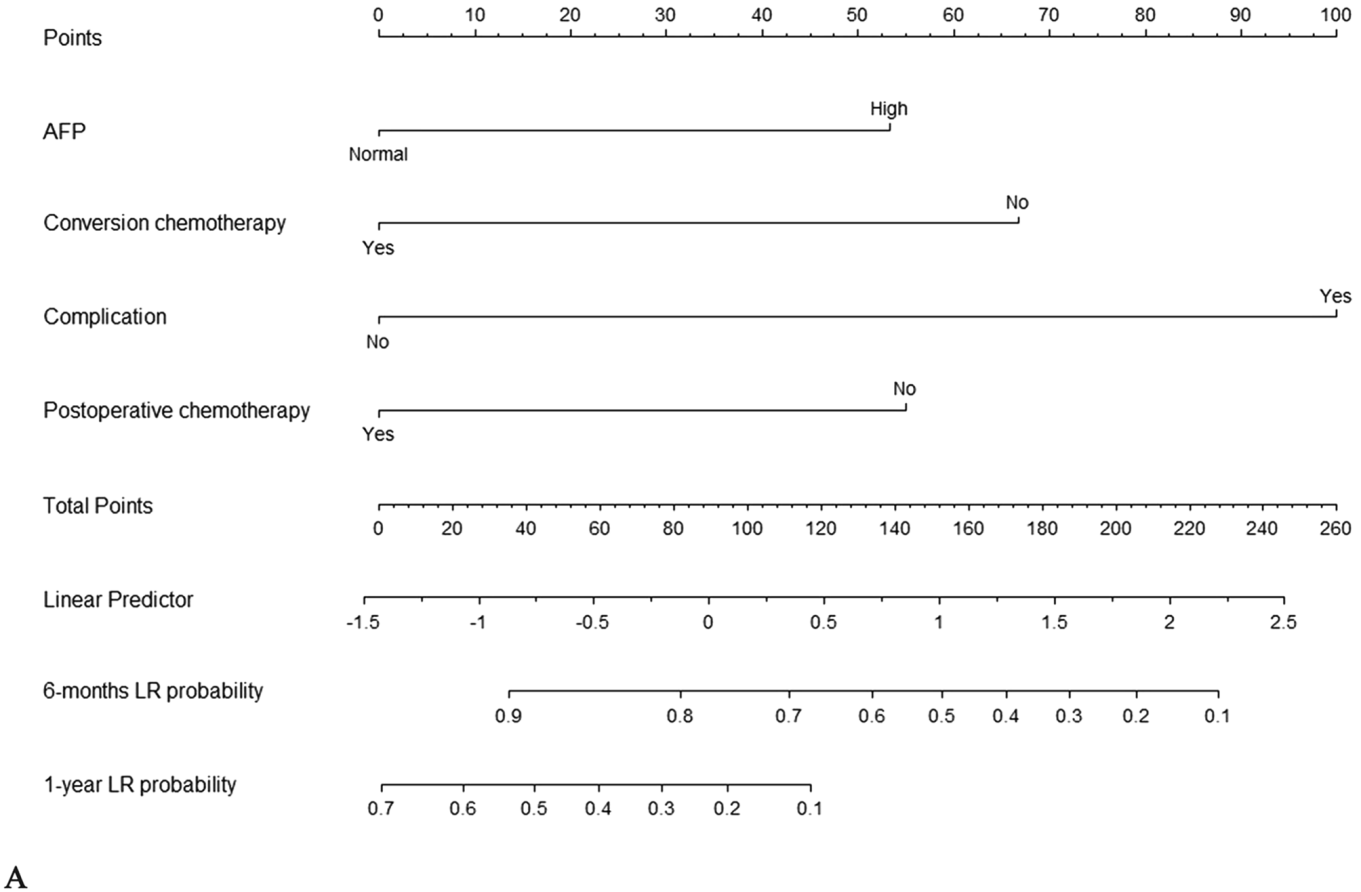

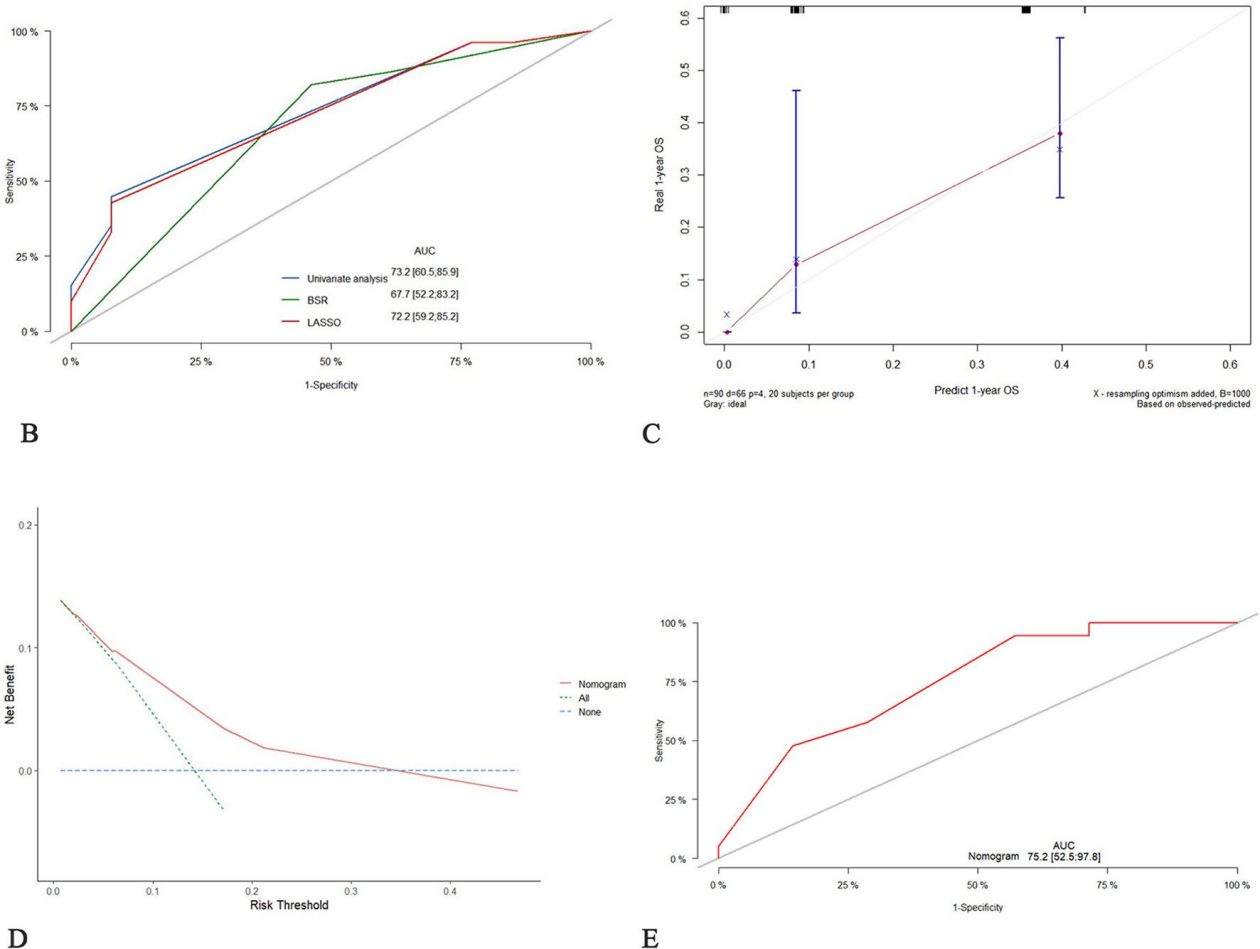
Development and performance of the nomogram. **A** Nomogram based on prognostic factors. **B** The ROC of three different Cox regression models. **C** 1-year calibration curves. **D** Decision curve of the nomogram. **E** The ROC of the nomogram in test cohort

Three statistical methods were used to analyze the clinical prognostic factors and treatment options, and Cox regression multivariate analysis was performed after screening. Four independent risk factors (*p* < 0.05) were identified: alpha-fetoprotein (AFP), HIPEC complications, conversion chemotherapy, and postoperative chemotherapy (Table 2). This four-factor regression model with the smallest AIC value (437.99) and the largest AUC value (0.732, 95% CI 0.605–0.859) (Fig. 2B) was superior to the model constructed by BSR and Lasso regression analysis (AIC: 449.75; 440.57) (Table 3, Fig. S1). We constructed a nomogram model according to the selected variables using a multivariate Cox analysis model wherein each patient’s total weighted score was calculated, and the corresponding score was obtained (Fig. 2A). Figure 2C shows the 1-year survival calibration curves for the training set. The decision curves in Fig. 2D shows that the predictive threshold of the nomogram is high. The test group was validated with an AUC of 0.752 (95% CI 0.525–978) and good predictive performance (Fig. 2E, Fig. S2).

**Table 2:** Cox regression analysis of the training cohort of patients by preoperative and intraoperative variables

Characteristics	Group	HR CI 95	*p* value
Sex	Male	Ref.	
	Female	0.6 (0.35–1.01)	0.057
Chronic disease	No	Ref.	
	Circulatory system disease	1.15 (0.49–2.71)	0.752
	Diabetes	1.86 (0.45–7.8)	0.394
	Others	1.18 (0.42–3.28)	0.754
Family history	No	Ref.	
	Yes	1.33 (0.41–4.26)	0.635
Pathology	Well-moderately differentiated adenocarcinoma	Ref.	
	Poorly differentiated adenocarcinoma	1.68 (0.6–4.7)	0.325
	Signet-ring cell carcinoma	0.65 (0.2–2.1)	0.468
Tumor site	Upper	Ref.	
	Middle	1.07 (0.5–2.3)	0.855
	Lower	1.23 (0.6–2.5)	0.573
cT	T3	Ref.	
	T4a	0.86 (0.37–2.01)	0.725
	T4b	0.94 (0.34–2.64)	0.91
Primary lesion size	2–5 cm	Ref.	
	5–10 cm	0.89 (0.49–1.61)	0.694
cN	N0-1	Ref.	
	N2	0.67 (0.31–1.45)	0.307
	N3	0.81 (0.38–1.7)	0.573
cM	P1	Ref.	
	M1	1.12 (0.34–3.64)	0.853
Borrmann classification	II	Ref.	
	III	0.78 (0.3–2.01)	0.603
	IV	0.91 (0.35–2.39)	0.848
Vessel carcinoma embolus	No	Ref.	
	Yes	1.24 (0.5–3.11)	0.642
Nerve invasion	No	Ref.	
	Yes	1.17 (0.66–2.07)	0.593
CEA	Normal	Ref.	
	High	1.18 (0.68–2.04)	0.548
CA199	Normal	Ref.	
	High	1 (0.61–1.65)	0.998
**AFP**	**Normal**	**Ref.**	
	**High**	**2.56 (1.31–5)**	**0.006**
CA724	Normal	Ref	
	High	1.61 (0.98–2.63)	0.061
**Ca125**	**Normal**	**Ref.**	
	**High**	**2.27 (1.32–3.91)**	**0.003**
Hemoglobin	> 120 g/L	Ref.	
	100–120 g/L	0.84 (0.48–1.46)	0.528
	70–100 g/L	0.91 (0.38–2.16)	0.825
	< 70 g/L	3.72 (0.5–27.98)	0.201
Leukopenia	Normal	Ref.	
	Low	1.15 (0.41–3.19)	0.793
	High	0.91 (0.36–2.29)	0.844
Thrombocytopenia	Normal	Ref.	
	Low	0.69 (0.29–1.62)	0.393
	High	1.38 (0.67–2.86)	0.384
PCI	< 7	Ref.	
	7–14	1.38 (0.59–3.21)	0.453
	> 14	1.13 (0.54–2.34)	0.745
Targeted therapy	No	Ref.	
	Yes	0.66 (0.34–1.27)	0.214
Immunotherapy	No	Ref.	
	Yes	0.47 (0.14–1.53)	0.21
**Conversion chemotherapy**	**No**	**Ref.**	
	**Yes**	**0.35 (0.14–0.88)**	**0.026**
**Complication**	**No**	**Ref**	
	**Yes**	**3.94 (1.22–12.75)**	**0.022**
Obstruction	No	Ref	
	Part	1.01 (0.55–1.84)	0.974
	Total	0.71 (0.38–1.34)	0.29
**Postoperative chemotherapy**	**No**	**Ref.**	
	**Yes**	**0.47 (0.28–0.81)**	**0.006**
BMI	BMI	1.04 (0.95–1.14)	0.406
Neutrophil to lymphocyte ratio	Neutrophil to lymphocyte ratio	0.99 (0.94–1.05)	0.848
Cycles	Cycles	0.87 (0.69–1.09)	0.223

Bold indicates *p* < 0.05

**Table 3:** Multivariate Cox regression models

Models of different statistical Analysis	Factors	HR (CI 95%)	*p* value	AIC	Likelihood ratio test	AUC (CI 95%)
**Univariate analysis**	AFP (normal)	Ref.		437.99	24.18	0.732 (0.605–0.859)
	AFP (high)	2.35 (1.19–4.63)	0.014			
	Complication (no)	Ref.				
	Complication (yes)	4.94 (1.49–16.4)	0.009			
	Conversion chemotherapy (no)	Ref.				
	Conversion chemotherapy (yes)	0.34 (0.13–0.88)	0.026			
	Postoperative chemotherapy (no)	Ref.				
	Postoperative chemotherapy (yes)	0.41 (0.23–0.72)	0.002			
Best Subsets Regression (BSR)	Pathology (well-moderately differentiated adenocarcinoma)	Ref.		449.75	8.42	0.677 (0.522–0.832)
	Pathology (poorly differentiated adenocarcinoma)	1.68 (0.59–4.70)	0.325			
	Pathology (signet-ring cell carcinoma)	0.65 (0.19–2.09)	0.468			
Least absolute shrinkage and selection operator (Lasso)	AFP (normal)	Ref.		440.57	19.6	0.722 (0.592–0.852)
	AFP (high)	2.20 (1.12–4.32)	0.021			
	Conversion chemotherapy (no)	Ref.				
	Conversion chemotherapy (yes)	0.33 (0.13–0.85)	0.022			
	Postoperative chemotherapy (no)	Ref.				
	Postoperative chemotherapy (yes)	0.43 (0.25–0.75)	0.003			

### Surgical benefit as predicted by the nomogram

**Fig. 3:**
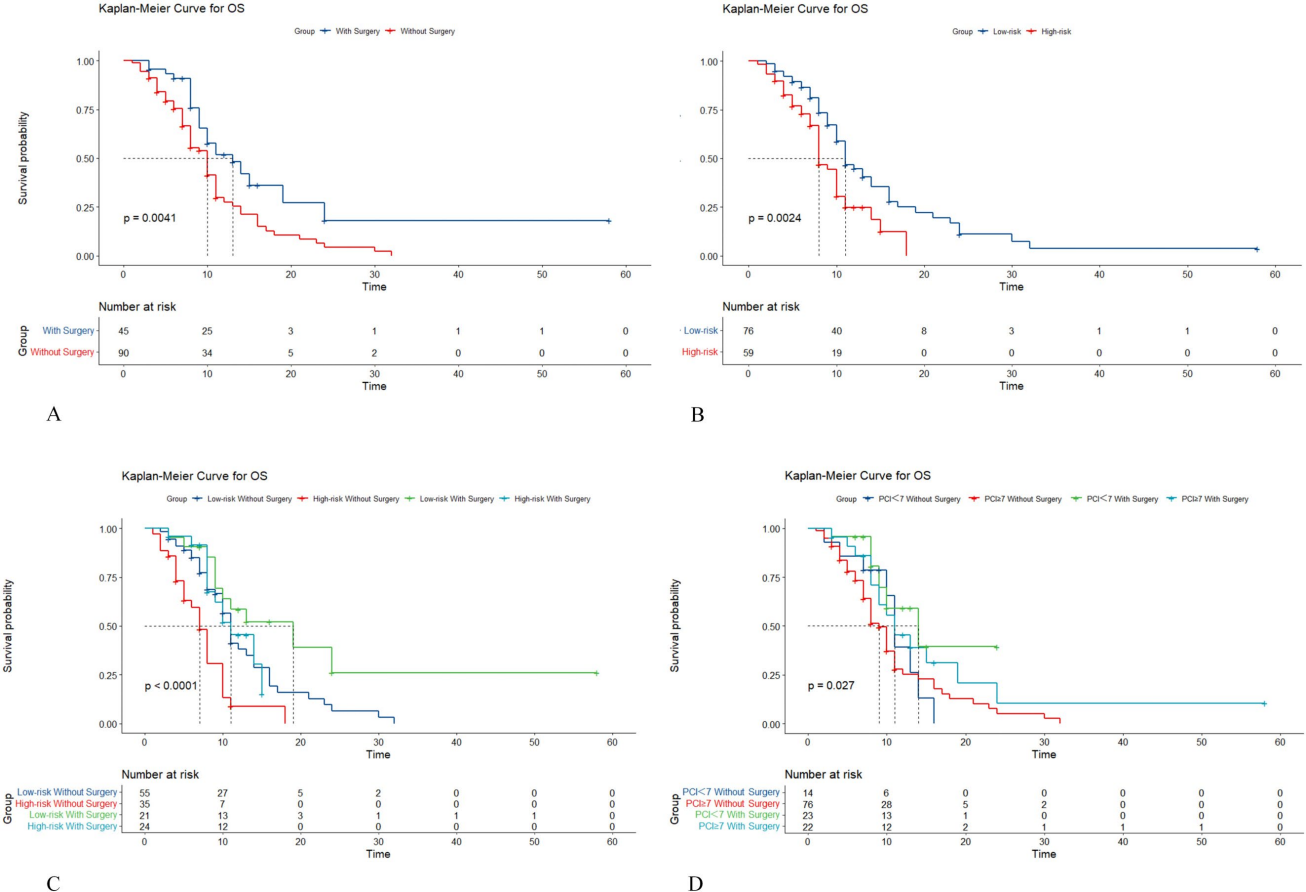
Kaplan–Meier Curve for OS. **A** OS of Low-risk and High-risk patients. **B** OS of patients with or without surgery. **C** OS of four groups. **D** OS of PCI < 7 and PCI ≥ 7 (with or without surgery)

Based on the ROC results of the model, the predictive cut-off value was determined to be 67 points. Based on this model and CRS, patients were divided into four groups: high-risk without surgery, low-risk without surgery, high-risk with surgery, and low-risk with surgery. In total population, the median survival time was 11 months for low-risk patients and 8 months for high-risk patients, with a statistically significant difference (*p* = 0.002). In the group that did not undergo surgery, the median survival time was 7 months in the high-risk group and 11 months in the low-risk group. In the surgery group, the median survival time was 11 months in the high-risk group and 19 months in the low-risk group. This difference was found to be statistically significant (*p* < 0.0001). However, it is worth noting that all patients in the high-risk group died within 2 years, while two patients in the low-risk group who underwent surgery survived for more than 2 years and are still alive as of the writing of this paper. Additionally, The results showed that among patients without surgery, median survival times were 11 vs 9 months for patients with PCI < 7 and PCI ≥ 7. Among patients with surgery, median survival times were 14 vs 11 months for patients with PCI < 7 and PCI ≥ 7, respectively. The differences were also statistically significant (*p* < 0.027) (Fig. 3).

## Discussion

Some studies have conducted a multifactorial analysis of gastric cancer patients with peritoneal metastasis and have established nomograms to evaluate the mortality risk of these patients. However, the analyses on the risk of mortality and the benefit of surgery in patients with gastric cancer who undergo HIPEC is still lacking. In this study, a nomogram was used for the first time to classify the risk of peritoneal metastasis in gastric cancer patients who received HIPEC. The predictive model, which includes four clinical factors and treatment otions, can accurately distinguish patients with a high risk of death from those with a low risk of death and may be used to guide the treatment plan of patients.

In the baseline characteristics of the patients, we found that the nodal stage was earlier and PCI was significantly lower in the population of patients with surgery. This suggests that nodal status and PCI are important indicators by which doctors decide whether to operate. Such biases occurred within our expectations. It is precisely because of this bias that the analysis of surgical benefit in patients undergoing HIPEC has become very difficult. However, in the present study, we cleverly avoided the effects of these two factors on the results by constructing a model. It was also found that the effects of these two factors on patients might be influenced by the four factors mentioned in the research. This is an important discovery. It implies that nodal metastasis and a high PCI may not be absolute contraindications to CRS.

In multivariate analysis, we identified four independent risk factors that were significantly associated with mortality. We found that elevated AFP was significantly associated with shorter patient survival, possibly because increased AFP often suggests that the patients have other undetected distant subclinical metastases, including liver metastases, which may affect overall survival. At the same time, previous studies have shown that the technique of HIPEC is relatively mature, so the possibility of complications in normal patients is very low [6]. If complications occur during HIPEC treatment, it may indicate that the cancers are more severe or the patient may have relatively hidden perforation, hemorrhage, and poor nutritional status before treatment. These problems may lead a poor prognosis.

Chemotherapy remains the standard of the treatment of patients with gastric cancer and peritoneal metastasis. Standardized conversion chemotherapy or postoperative chemotherapy is of positive significance for patient survival. This finding is similar to the results of previous studies. For patients with good physical and economic conditions, perioperative chemotherapy should be actively administered as this can increase the possibility of surgery and improve the prognosis of patients. If the patient is unable to undergo standard systemic therapy for various reasons, the benefits of surgery will be reduced.

Previous studies have shown that the median survival time after CRS combined with HIPEC is significantly prolonged to 18.8 months. However, whether all patients with peritoneal metastases can benefit from surgery remains controversial [12]. In the current study, high- and low-risk populations were statistically analyzed. These results suggest that surgery can prolong the survival of patients who undergo HIPEC. It is worth noting that in the high-risk population, all patients died within 2 years, regardless of whether surgery was performed, and surgery only prolonged survival by 4 months. However, for low-risk patients, aggressive surgical treatment can significantly prolong survival by up to 7 months, and some patients can achieve long-term survival. Therefore, we believe that surgery combined with HIPEC should be actively performed to improve the prognosis of patients with a low clinical risk. For patients with peritoneal metastasis with a high risk of clinical death, surgical treatment can be attempted while ensuring standard systemic treatment, but the wishes of patients and their families should be taken into consideration, and the judgment should be made carefully according to the individual clinical situation. Finally, we analyzed the prognostic impact of PCI in both groups of patients. The results showed that the PCI was indeed able to influence the patient's prognosis. However, in both surgery and no-surgery groups, the prolonged survival time of patients with PCI < 7 did not exceed 3 months. The results of the model are better than those of PCI.

The training and test group populations in this study were not the same as those in previous studies. Because CRS + HIPEC is not the best treatment recommendation for patients with gastric cancer and peritoneal metastasis in China, very few peritoneal metastasis patients underwent CRS. Objective statistical results are difficult to achieve if using traditional study methods. So we decided to construct the model by patients who had been only treated with HIPEC in guiding the choice of surgery. After consulting with statistical and clinical experts, the study design achieved the preset research purpose and met the statistical requirements. Although the sample size of this study was not large enough to establish a very accurate model for predicting surgical benefits, this study showed an important clinical suggestion: CRS may be the optimal treatment for all peritoneal metastasis gastric cancer patients treated by HIPEC, especially low-risk patients, which can further improve the probability of long-term survival. In the future, we will continue to expand the sample size and develop more accurate prediction models to predict the survival benefits of patients undergoing surgery.

## Conclusion

The nomogram constructed using the four factors identified in the study can accurately predict the risk of death in gastric cancer patients with peritoneal metastasis treated with HIPEC. CRS can prolong the survival time of these patients. In particular, for low-risk patients, the benefits of aggressive surgical treatment were more pronounced.

## Supplementary Information

Below is the link to the electronic supplementary material.

**Figure S1:** Figures of BSR (**A**, **B**); Figures of LASSO regression (**C**, **D**) (JPG 268 KB)

**Figure S2:** The time-dependent ROC curves of training cohort (**A**); the time-dependent ROC curve of Test cohort (**B**) (JPG 256 KB)

Supplementary file3 (DOC 353 KB)

## Data Availability

The data are available from the corresponding author on reasonable request.
