# Supplementary figures and images for: Prediction of surgical benefit in gastric cancer patients with peritoneal metastasis treated with hyperthermic intraperitoneal chemotherapy
Source: Updates Surg. 2024 Oct 4;76(7):2663–74. doi: 10.1007/s13304-024-01989-y (PMC11602788; doi:10.1007/s13304-024-01989-y)

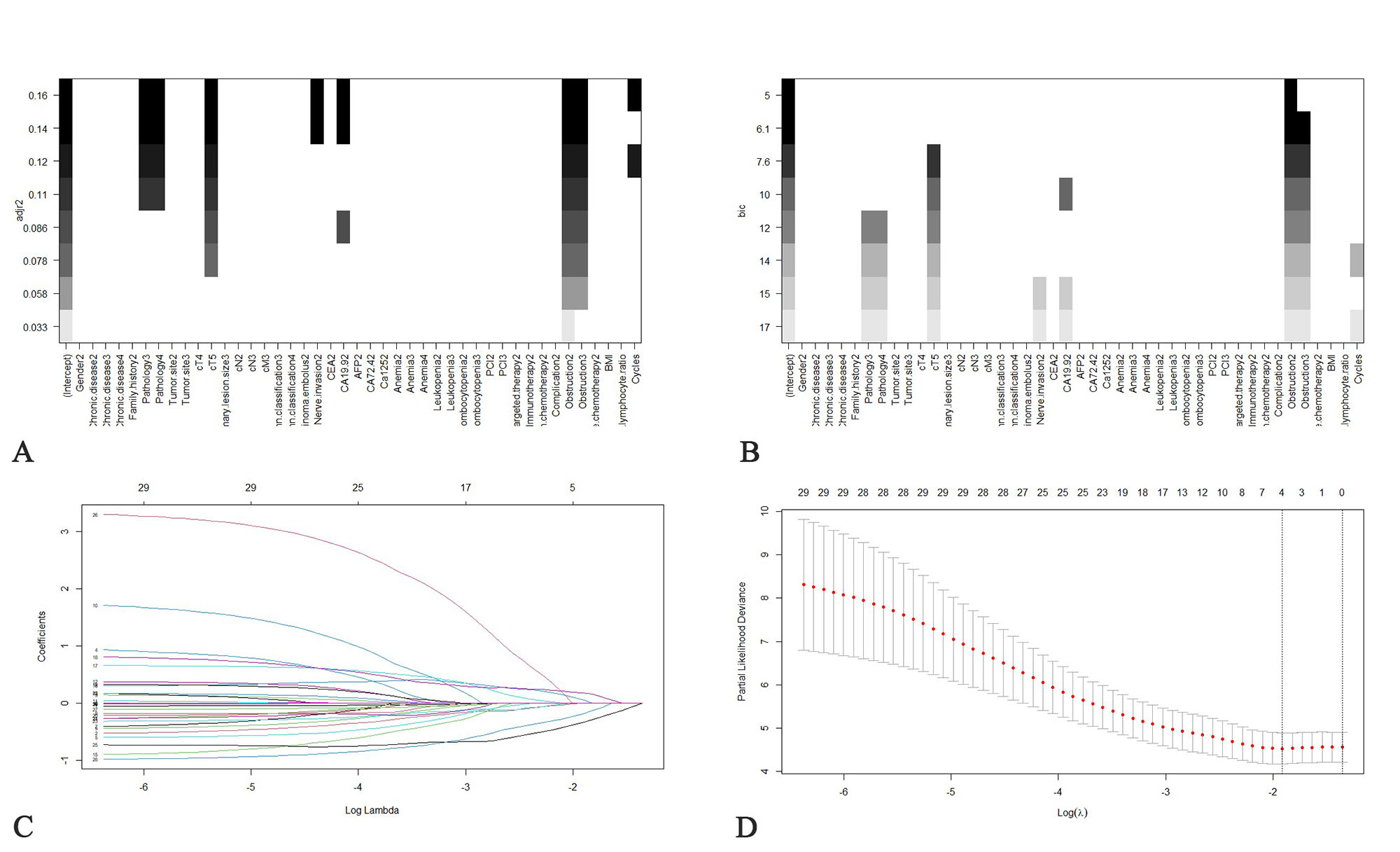

Supplement: Supplementary file 1 — Figure S1: Figures of BSR (A, B); Figures of LASSO regression (C, D) (JPG 268 KB) [file 13304_2024_1989_MOESM1_ESM.jpg]

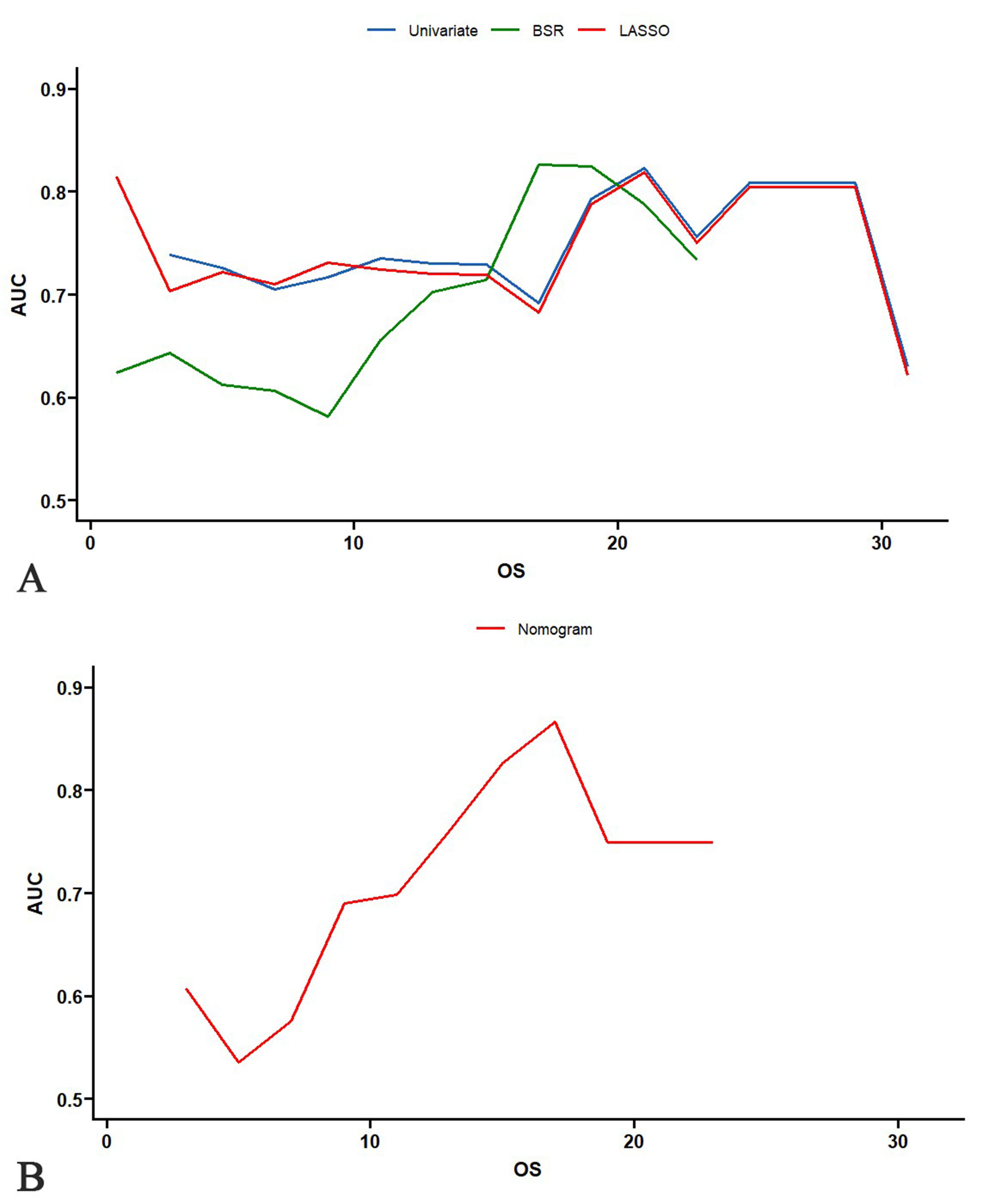

Supplement: Supplementary file 2 — Figure S2: The time-dependent ROC curves of training cohort (A); the time-dependent ROC curve of Test cohort (B) (JPG 256 KB) [file 13304_2024_1989_MOESM2_ESM.jpg]
