# Supplementary material for: Prediction of surgical benefit in gastric cancer patients with peritoneal metastasis treated with hyperthermic intraperitoneal chemotherapy
Source: Updates Surg. 2024 Oct 4;76(7):2663–74. doi: 10.1007/s13304-024-01989-y (PMC11602788; doi:10.1007/s13304-024-01989-y)
Supplement: Supplementary file 3 — Supplementary file3 (DOC 353 KB) [file 13304_2024_1989_MOESM3_ESM.doc]

| **Table S1: Clinicopathologic characteristics of the patients (total).** | | | |
| --- | --- | --- | --- |
| Category | Training cohort  (without surgery) | Test cohort  (with surgery) | p |
|  | N=90 | N=45 |  |
| **Age (mean (SD))** | 53.856 (11.414) | 54.956 (10.445) | 0.5883 |
| **BMI (mean (SD))** | 22.310 (3.100) | 21.867 (3.761) | 0.4678 |
| **Sex (%)** |  |  | 0.2937 |
| Male | 56 (62.22) | 23 (51.11) |  |
| Female | 34 (37.78) | 22 (48.89) |  |
| **Pathology (%)** |  |  | 0.7581 |
| Well-moderately differentiated adenocarcinoma | 4 (4.44) | 1 (2.22) |  |
| Poorly differentiated adenocarcinoma | 68 (75.56) | 36 (80.00) |  |
| Signet-ring cell carcinoma | 18 (20.00) | 8 (17.78) |  |
| **Tumor site (%)** |  |  | 0.0633 |
| Upper | 15 (16.67) | 3 (6.67) |  |
| Middle | 29 (32.22) | 23 (51.11) |  |
| Lower | 46 (51.11) | 19 (42.22) |  |
| **cT (%)** |  |  | 0.9587 |
| T3 | 9 (10.00) | 5 (11.11) |  |
| T4a | 70 (77.78) | 34 (75.56) |  |
| T4b | 11 (12.22) | 6 (13.33) |  |
| **cN (%)** |  |  | <0.0001 |
| N0-1 | 12 (13.33) | 12 (26.67) |  |
| N2 | 35 (38.89) | 33 (73.33) |  |
| N3 | 43 (47.78) | 0 (0.00) |  |
| **cM (%)** |  |  | 0.0498 |
| M0 | 0 (0.00) | 2 (4.44) |  |
| P1 | 86 (95.56) | 43 (95.56) |  |
| M1 | 4 (4.44) | 0 (0.00) |  |
| **Primary lesion size (%)** |  |  | 0.1829 |
| 2-5cm | 20 (22.22) | 6 (13.33) |  |
| 5-10cm | 70 (77.78) | 38 (84.44) |  |
| >10cm | 0 (0.00) | 1 (2.22) |  |
| **Chronic disease (%)** |  |  | 0.1523 |
| No | 74 (82.22) | 42 (93.33) |  |
| Circulatory system disease | 8 (8.89) | 0 (0.00) |  |
| Diabetes | 3 (3.33) | 2 (4.44) |  |
| Others | 5 (5.56) | 1 (2.22) |  |
| **Borrmann classification (%)** |  |  | 0.3862 |
| Ⅱ | 7 (7.78) | 1 (2.22) |  |
| Ⅲ | 49 (54.44) | 24 (53.33) |  |
| Ⅳ | 34 (37.78) | 20 (44.44) |  |
| **PCI (%)** |  |  | <0.0001 |
| <6 | 14 (15.56) | 23 (51.11) |  |
| 7-14 | 19 (21.11) | 13 (28.89) |  |
| >14 | 57 (63.33) | 9 (20.00) |  |
| **Conversion chemotherapy (%)** |  |  | 0.1189 |
| No | 82 (91.11) | 36 (80.00) |  |
| Yes | 8 (8.89) | 9 (20.00) |  |
| **Postoperative chemotherapy (%)** |  |  | 0.4107 |
| No | 30 (33.33) | 19 (42.22) |  |
| Yes | 60 (66.67) | 26 (57.78) |  |
| **Targeted therapy (%)** |  |  | 0.6996 |
| No | 74 (82.22) | 35 (77.78) |  |
| Yes | 16 (17.78) | 10 (22.22) |  |
| **Immunotherapy (%)** |  |  | 0.2723 |
| No | 86 (95.56) | 40 (88.89) |  |
| Yes | 4 (4.44) | 5 (11.11) |  |
| **Complication (%)** |  |  | 0.0105 |
| No | 87 (96.67) | 37 (82.22) |  |
| Yes | 3 (3.33) | 8 (17.78) |  |
| **Obstruction (%)** |  |  | 0.3113 |
| No | 39 (43.33) | 23 (51.11) |  |
| Part | 27 (30.00) | 8 (17.78) |  |
| Total | 24 (26.67) | 14 (31.11) |  |
| **OS** | 10（8-11） | 13（10-NA） | 0.0221 |

| **Table S2: Complications within 90 days (total).** | | |
| --- | --- | --- |
| Category | Training cohort  (without surgery) | Test cohort  (with surgery) |
|  | N=3 | N=8 |
| **Abdominal infection** | 3 | 5 |
| Grade 1 | 1 | 3 |
| Grade 2 | 1 | 2 |
| Grade 3 | 0 | 0 |
| Grade 4 | 1 | 0 |
| Grade 5 | 0 | 0 |
| **Electrolyte disturbance** | 0 | 2 |
| Grade 1 | 0 | 1 |
| Grade 2 | 0 | 1 |
| Grade 3 | 0 | 0 |
| Grade 4 | 0 | 0 |
| Grade 5 | 0 | 0 |
| **Delirium** | 0 | 1 |
| Grade 1 | 0 | 0 |
| Grade 2 | 0 | 1 |
| Grade 3 | 0 | 0 |
| Grade 4 | 0 | 0 |
| Grade 5 | 0 | 0 |
